# Effects of Conservative Tillage and Nitrogen Management on Weed Seed Bank after a Seven-Year Durum Wheat—Faba Bean Rotation

**DOI:** 10.3390/plants7040082

**Published:** 2018-09-30

**Authors:** Mariano Fracchiolla, Anna Maria Stellacci, Eugenio Cazzato, Luigi Tedone, Salem Alhajj Ali, Giuseppe De Mastro

**Affiliations:** 1 Department of Agricultural and Environmental Science, University of Bari, 70125 Bari, Italy; mariano.fracchiolla@uniba.it (M.F.); luigi.tedone@uniba.it (L.T.); salem_grb@yahoo.com (S.A.A.); giuseppe.demastro@uniba.it (G.D.M.); 2 Department of Soil, Plant and Food Sciences, University of Bari Aldo Moro, 70125 Bari, Italy; annamaria.stellacci@uniba.it

**Keywords:** stepwise discriminant analysis, conservation agriculture, weeds, sod seeding, fertilization

## Abstract

Conservative agriculture includes a range of management strategies with low energy inputs such as no-tillage, minimum tillage, and low application of fertilizers. Weed flora in arable fields is strictly affected by agronomic practices such as tillage and fertilization management. This study was conducted seven years after the beginning of a long-term—durum wheat–faba bean—rotation. It analyzes the combined effects on the soil seed bank of three different tillage systems (conservative, reduced, and conventional tillage) and two levels of nitrogen fertilization. The effects were investigated both using stepwise discriminant analysis and analysis of variance in order to find statistical differences among main factors and their interactions. The seed bank of *Conyza canadensis*, *Papaver rhoeas*, *Solanum nigrum*, *Fallopia convolvulus*, and *Fumaria officinalis* was higher in conservative or reduced tillage plots. The magnitude of the response to nitrogen supply varied among weed species. *Conyza canadensis* seemed to be favored by low nitrogen supply, whereas *Sinapis arvensis* by higher doses of nitrogen. *Anagallis arvensis* showed the lowest seed bank in conventionally tilled plots, without distinction of nitrogen supply. The results suggest that different tillage systems and, to a lesser extent, different nitrogen supply, produce changes in the seed bank size and composition, along the soil profile.

## 1. Introduction

Conservation agriculture has gained popularity in many agriculture systems all over the world. It is defined by FAO as an “approach to managing agro-ecosystems for improved and sustained productivity, increased profits and food security while preserving and enhancing the resource base and the environment” [1]. Conservation agriculture promotes, mainly, minimal soil disturbance and low application of chemical inputs such as fertilizers and pesticides, therefore reduction of energy inputs. Minimal soil disturbance plays a pivotal role because it reduces the consumption of fossil fuels, avoids soil erosion, maintains soil physical and biological health [2]. In the same way as for tillage, also, nitrogen applications need to be rationally managed because N affects environmental performance of agriculture practices by increasing the emissions of greenhouse gases [3] and the risk of groundwater contamination. Moreover, N coming from agricultural systems is biologically active in soil and water or chemically active in atmosphere; therefore, it can be detrimental for biological diversity, air, and water [4].

Weed flora, in terms of both weed density and species composition, in arable fields is strictly affected by agronomic practices such as tillage and fertilization management.

The distribution of weed seeds within the soil is modified by tillage, therefore germination and emergence rate can be strongly affected [5]. Specifically, one of the main characteristic that affects weed infestation according to tillage is the different ability of species to germinate from increasing soil depths. Benvenuti et al. [6] studied the effects of seed burial depth on seedling emergence of several species. They found marked differences among the species, although the emergence rate always decreased with increasing soil depth.

Consequently, different species show different response to the tillage systems and their spreading can be promoted or inhibited in relation to their ecophysiological characteristics [7,8].

Differently from conventional tillage, conservation agriculture practices include a range of tillage regimes, such as no-tillage (direct drilling) and minimum tillage (shallow tillage), that avoid soil inversion. Consequently, it is reasonable to suppose that weed community is affected very differently by these management systems [9], as shown by many previous studies.

Armengot et al. [10] reported data about seven trials in different climatic regions of Europe; they observed an increasing trend in weed richness under reduced tillage compared with conventional tillage as well as strong effects of the type of tillage on the weed community composition. In addition, authors found also differences among functional traits of weeds (such as seed weight, flowering span, and height) that can make individuals able to fit to different conditions.

Froud-Williams et al. [11] analyzed the effects of reduced-cultivation systems on weeds in cereals. The authors highlighted a change of existing weed flora; particularly, some species previously unimportant may become prevalent (e.g., *Bromus* spp.) and other species characteristic of arable lands are expected to decline.

On winter cereals, Scherner et al. [12] studied the effects of different tillage systems on emergence dynamics of annual grass weeds. The emergence of weed seedlings varied between the tillage systems with a higher total emergence observed under direct drilling, followed by presowing tine cultivation and plowing. Also Santín-Montanyá et al. [13], observed after several years, in cereal crops, the increasing of weed abundance in conservation tillage systems.

Field studies, conducted to evaluate the effects of tillage systems on weed density and species composition in rotations including wheat, soybean, and maize, showed a rapid change of weed spectrum in no-tillage plots [14].

Tillage systems produce also a differentiation of the soil seed bank that is also related to the density of actual flora. At the end of 10 years of continuous spring barley, Conn [15] found a total seed density greater under no-till than under other tillage treatments. In several crop rotations (continuous corn, corn–soybean, and corn–oats–hay) seed density was found highest in no-tillage and declined as tillage intensity increased [16]. Similar results are reported also by Legere et al. [17] in different crop rotations (2-year barley–red clover followed by 4-year barley–canola–wheat–soybean). Feldman et al. [18] conducted a study on wheat grown with different tillage systems (moldboard plough, disk chisel, and no-tillage), concluding that less soil disturbance causes a larger and more diverse seed bank.

Each weed species does not have the same affinity to the tillage systems. Basing on several bibliographic data, Forcella and Burnside [7] report a list of species and their response to conventional, reduced, or no-tillage. For example, the list shows that *Setaria faberi* is inhibited by conventional tillage and promoted by reduced tillage and no-tillage; *Chenopodium album* is indifferent; *Amaranthus retroflexus* and *Abutilon theophrasti* are promoted by conventional tillage; *Cirsium arvense* is inhibited by conventional tillage and promoted by no-tillage.

Moreover, in *Zea mays* L. Swanton et al. [19] found that *Chenopodium album* and *Amaranthus retroflexus* were associated with conventional tillage and *Digitariasanguinalis* with no-tillage. Occurrence of *Conyzacanadensis* has usually been associated with no tillage systems [20,21].

A different affinity of wild species to tillage results also in orchards, as shown by Fracchiolla et al. [22]; authors found that *Poaceae* species (*Bronus sterilis*, *Avenas terilis* and *Hordeum murinum*) are significantly associated with no-tillage systems such as the application of foliar herbicides or mowing.

Weed community and soil seed bank can be affected also by nitrogen content as a consequence of different amounts and types of fertilizers [23]. The response of each species can be very different as shown by many authors such as Blackshaw [24] and Sweeney [25]. Further, Pysek and Leps [26] suggest that weed community is influenced by both a direct effect of the fertilizer and indirect effects such as the increased competition with the crop. To this regard, time of fertilization supply, in relation to time of weed emergence, plays a crucial role.

Nitrogen can also play a role in regulating seed germination; for example, it can stimulate or inhibit germination of dormant seeds as shown by Goudey et al. [27] for *Sinapis arvensis*.

Moreover, nitrogen fertilizers and tillage systems can interact in modifying weed infestation as shown in barley (*Hordeum vulgare*) by O’Donovan et al. [28]; particularly, authors report that *Setaria viridis* was associated with low nitrogen rate and conventional tillage.

Therefore, the extensive literature review concludes that weed species richness, seed bank abundance, and diversity are dependent on disturbance levels of the soil and fertilization rate. The effects are also influenced by the interaction with other factors such as the environmental conditions and crop species [9]. The adoption of conservation tillage provides different environments for weed germination, emergence, growth, and competition by altering physical and chemical properties of soil [18]. Fertilizers, especially nitrogen, interact with the different tillage systems influencing weed germination, emergence, growth, and reproduction.

In investigating the effects of different agronomic managements, and their interactions, on weed density and species composition, many variables (weed species) have to be simultaneously taken into account and analyzed. To this aim, a crucial role in interpreting the results of field monitoring is played by the adoption of multivariate analysis techniques. Multivariate methods, and in particular discriminating techniques, allow to easily summarize the effects observed and extract the main behaviors in relation to the treatments compared [29]. In particular, stepwise discriminant analysis (SDA) is a feature selection technique which allows the identification of the variables enabling maximum discrimination among the treatments (i.e., classes) compared.

Knowledge of long-term effects on weed species and communities in agroecosystems will provide useful information to farmers about possible future scenarios that can be encountered as a consequence of changes in field management.

For all these reasons, this study evaluates, in a fava bean–durum wheat rotation started in 2008, the combined effects on the seed bank of different tillage systems and nitrogen fertilization levels.

## 2. Results

### 2.1. Total Number of Species and Seeds in the Seed Bank

A total number of 21 species was found in the soil seed bank, belonging to 13 botanical families. Among all the species, 11 of them, in the experimental area, have the emergence phase typically in Spring or Summer i.e., between May and July, whereas 10 emerge in Autumn or Winter (October–January) (Table 1).

Regarding total number of seeds m^−2^, only the tillage systems caused significant differences (Table 2) as a consequence of the intensity of soil disturbance; particularly, the number of seeds was the highest in the Conservative Tillage (SS), followed by Reduced Tillage (RT), and Conventional Tillage (CT). The total number of seeds of the species emerging in spring or summer showed the same trend. The total number of seeds emerging in autumn/winter was higher in SS than in CT plots; for these species, the number of seeds m^−1^ was higher also in the plots fertilized with 90 kg ha^−1^ of N.

Significant interactions were found within soil depth and tillage for total number of seeds and number of seeds of autumn/winter species. Particularly, both these data were the highest in the layer 0 to 0.20 m of the SS plots (data not shown).

### 2.2. Seed Bank Composition

#### 2.2.1. Stepwise Discriminant Analysis

Stepwise Discriminant Analysis allowed a first and overall screening of the effects of the treatments compared on weed seed bank.

Specifically, in the analysis carried out on the upper soil layer (0–0.20 m), SDA identified *Anagallis arvensis*, *Conyza canadensis*, and *Heliotropium europaeum* as the weed species most able in discriminating both different soil tillage and N supply (T × N) (Table 3 (a)).

By investigating in detail the effect of the three soil managements (T), together with *Conyza canadensis*, *Papaver rhoeas*, *Solanum nigrum*, and *Fallopia convolvulus* were selected as the species most affected by the treatments compared in the shallower soil layer (Table 3 (b)). Finally, a greater number of species, including also *Sinapis arvensis*, *Fumaria officinalis*, *Chenopodium album*, and *Phalaris paradoxa*, was identified as the most discriminating the different N supplies (Table 3 (c)).

When the analysis was carried out on the 0.20 to 0.40 m soil layer, *Conyza canadensis* for the T × N interaction and N, together with *Papaver rhoeas* and *Phalaris paradoxa*, respectively for T x N and T, confirmed the behavior observed in the shallower soil layer (0–0.20 m) showing to be among the most sensitive species with regard to soil and N fertilization management (Table 4 (a–c)).

In detail, *Conyza canadensis*, together with *Papaver rhoeas*, *Portulaca oleracea*, and *Phalaris paradoxa*, showed the highest discriminating capability in the T × N interaction; *Papaver rhoeas*, *Phalaris paradoxa*, and *Fumaria officinalis* showed to be most affected by the different soil tillage, *Conyzacanadensis* and *Sonchusoleraceus* by the different N supplies.

Finally, on the analysis performed on the whole layer investigated (0–0.40 m), SDA identified *Conyza canadensis* among the weed species most able in discriminating both the different soil managements (T) and the whole treatments studied (T × N interaction). *Conyzacanadensis* was indeed selected as the second and third species, respectively, with partial R-Squared values of 0.4781 (F = 14.65, *p* < 0.0001) and 0.7085 (F = 13.61, *p* < 0.0001).

*Anagallis arvensis* was also selected as first species for both the different soil managements (T) and the whole treatments studied (T × N interaction), with partial R-Squared values of 0.5667 (F = 21.58, *p* < 0.0001) and 0.9112 (F = 61.53, *p* < 0.0001), respectively.

#### 2.2.2. Analysis of Variance

Table 5 shows the main results of Analysis of Variance performed for each species recorded in the soil samples, both for the main factors and for their interactions.

As regard to effect of soil depth, number of seeds recorded in the 0 to 0.20 m layer was significantly higher for *Fallopia convolvulus*, *Galium aparine*, *Papaver rhoeas*, *Polygonum aviculare*, and lower only for *Veronicahederifolia*. For the other species, ANOVA did not show any significant difference.

Except for *Polygonum aviculare* and *Veronica hederifolia*, tillage showed to induce significant modifications of the seed bank of all the species considered. Number of seeds found in SS plots was the highest for *Conyza canadensis*, *Fallopiaconvolvulus*, *Galiumaparine*, *Heliotropiumeuropaeum*, *Papaver hybridum*, *Portulaca oleracea*, *Setaria viridis*, *Solanum nigrum*, and *Silybum marianum*. Number of seeds of *Anagallis arvensis*, *Fumaria officinalis*, and *Papaver rhoeas* was the highest in RT plots, whereas only *Phalaris paradoxa* showed the highest seed bank in the CT plots.

With regard to the effect of nitrogen, in the plots fertilized with 30 kg ha^−1^ of N, the number of seeds of *Anagallis arvensis*, *Conyza canadensis*, and *Solanum nigrum* was higher than in those fertilized with the greater dose. Opposite results were recorded for *Papaver hybridum*, *Portulaca oleracea*, and *Sinapis arvensis* whose seed bank was higher in the plots with 90 kg ha^−1^ of N.

Regarding interaction soil depth × tillage, both in the layer 0 to 0.20 m and 0.20 to 0.40 m, the number of seeds m^−2^ of *Conyza canadensis* in SS plots was higher than in CT plots. Seeds of *Fallopia convolvulus* and *Galium aparine* were found only in the SS plots at both layers of soils studied with a value significantly higher in the upper layer. *Papaver rhoeas* was found only in both layers of RT plots and in the layer 0 to 0.20 m of SS.

This interaction soil depth × nitrogen was significant only for *Conyza canadensis*, *Papaver rhoeas*, and *Polygonumaviculare*.

The interaction tillage × nitrogen confirmed some behaviors highlighted by SDA, in particular regarding *Anagallis arvensis* and *Portulaca oleracea*. Specifically, the lowest number of seeds of *Anagallis arvensis* was found in all CT plots and SS plots fertilized with 90 kg ha^−1^ of N. Seed bank of *Papaver rhoeas* was statistically higher in RT (at both fertilization doses) and in SS plots fertilized with 90 kg ha^−1^ of N than in CT plots at both fertilization levels. The number of seeds of *Portulaca oleracea* was higher in SS plots with 90 kg ha^−1^ of N than in CT plots with 30 kg ha^−1^ of N; in addition, seeds of this species were not found in the other plots. The highest number of seeds of *Setaria viridis* was found in the SS plots fertilized with 90 kg ha^−1^ of N. Seed bank of *Solanum nigrum* in SS plots with 30 kg ha^−1^ of N was higher than in the all the other plots.

As for soil depth × tillage × nitrogen, at both depths, number of seeds m^−2^ of *Conyza canadensis* was the highest in all the SS plots except for those fertilized with 90 kg ha^−1^ of N at the layer 0.20 to 0.40 m. Number of seeds of *Galium aparine* was the highest in the layer 0 to 0.20 m of SS plots fertilized with 90kg ha^−1^.

## 3. Discussion

Among all the species found in the study area, the results show clear evidence that some species seed banksare particularly affected by tillage systems and the level of nitrogen supply.

In the layer 0 to 0.20 m, tillage systems were discriminated overall by *Conyza canadensis*, *Papaver rhoeas*, *Solanumnigrum*, and *Fallopia convolvulus* whereas in the layer 0.20 to 0.40 m by *Phalaris paradoxa*, *Papaver rhoeas*, and *Fumaria officinalis*. Except for *Phalaris paradoxa* whose seed bank was higher in the deeper layer of conventionally tilled plots (moldboard plowing), the seed bank of all the above mentioned species appears promoted by no-tillage or, at least, reduced tillage.

*Conyza canadensis* seemed to be favored by low cultural inputs in terms of soil disturbance and nitrogen supply. Similar results are reported by other studies particularly regarding the spreading of this weed caused by “no-reverse” tillage systems such as no-tillage [30,31] or chisel plowing [32].

The magnitude of the response to nitrogen supply varied among weed species, as shown also by Blackshaw et al. [24]. In the upper layer, six species were able in discriminating the two different doses of nitrogen whereas only two in the deeper layer. Among these species, *Conyza canadensis* appears promoted by low nitrogen supply whereas *Sinapis arvensis* by the higher doses of nitrogen. This last species has been already reported as benefiting from increased nitrogen availability [33].

*Anagallis arvensis* clearly discriminated, as also highlighted by the SDA results, the whole treatments studied, showing the lowest seed bank in the conventionally tilled plots without distinction of depth or nitrogen supply. The seed bank of *Portulaca oleracea*, a nitrophilous species [34], appears to be promoted, at all the layers studied, by no-tillage and high levels of nitrogen supply.

On average, the effect of tillage system on most of the species found in the experimental field and on overall weed community was more marked than that of nitrogen supply.

Findings of our research are consistent with those reported by many authors [10,16,18]: reduced or no-tillage caused an increasing infestation of many weed species because of low soil disturbance. Of the species emerging in autumn or winter, the effect of nitrogen supply is more evident; it makes sense to hypothesize that these species are growing at the time of fertilizer distribution and therefore benefit directly from the increased availability of nitrogen.

## 4. Materials and Methods

Data were collected in a field trial located in Southern Italy (Policoro–Basilicata Region) at the experimental farm “E. Pantanelli” (University of Bari—40°10′19.70″ N; 16°39′5.05″ E).

The site is 15 m above sea level and characterized by subarid climate according to De Martonne classification [35], with average annual rainfall of 560 mm distributed mainly during autumn and winter, and temperature typical of Mediterranean climate.

The soil, according to the Canadian System of Soil Classification [36], is clay-loam with high content of organic matter (Table 6).

Since 2008, the field hosted a long-term—durum wheat–faba bean—rotation in a split-plot experimental design. Within the experiment field, an area was isolated and considered to investigate the effects of tillage system (Conservative tillage—SS; Reduced tillage—RT; Conventional tillage—CT) and nitrogen supply (low and high N rate) on weed seedbank size and composition (Table 7).

Specifically, the field had been divided into three strips (each of 30 × 80 m) managed with three different tillage systems (Table 7). Each strip had been subdivided into two plots (30 × 40 m) in which 43.8 or 131.4 kg of Urea (46%) (corresponding to 30 or 90 kg ha^−^¹ of N) were applied when durum wheat was grown. Fertilizer was applied at the end of February (at the end of tillering phase) and spread uniformly all over the plot.

Further details about conduction of the trial can be found in a past paper [3].

Wheat (*Triticum turgidum* L. var *durum*) cv *Iride* was sown at a rate of 200 kg ha^−1^, to obtain a density of 400 seeds m^−2^, whereas faba bean (*Viciafaba* var. *equina* Pers.) cv Prothabat 69 at a rate of 180 kg ha^−1^ to obtain a density of 55 seeds m^−2^. Both crops were sown in November and harvested in June.

Both in faba bean and durum wheat, weeds were chemically controlled applying the herbicides listed in Table 8.

### 4.1. Soil Sampling

Soil sampling was done in November 2015 (i.e., seven years after the beginning of the rotation) and before the preparation of the seedbed for the sowing of faba bean. Each of the six plots (three tillage systems × 2 N levels) was divided into three parts; in each of them, twenty soil cores of 0.40 m depth were randomly taken within a central area of 10 m × 20 m. A 2.3-cm diameter cylindrical steel probe was used, subdividing each core into two sub-cores of 0.20 m, corresponding to 0–0.2 m and 0.2–0.4 m depths. The twenty samples taken in each part were mixed to form a single sample that represented soil, randomly collected, and deriving from a surface of 8.306 × 10^−3^ m^2^.

### 4.2. Seed Bank Determination

For the determination of the seed bank, the direct observation of the plantlets emerging from each soil sample was applied [32,37]. Some species, for which it was not possible to identify at the early stages, were transplanted in pots and allowed to grow to be identified at older stages.

Soil samples were placed into a tray where the soil did not exceed 4 cm depth and was periodically wetted. Trays were kept in the greenhouse for 20 months, under such conditions as not to exceed an internal temperature of 35 °C, during the warmest periods.

In order to favor dormancy breakage, irrigation was suspended periodically for 15 days and samples were stirred before irrigation were resumed; four months before the end of the experiment, samples were irrigated with a solution containing 1000 mg L^−1^ of KNO_3_ to stimulate the germination of residual viable seeds. The plantlets emerging from each tray were identified, counted and removed. The data collected for each species were expressed in terms of number of seeds m^−2^ of soil [32,38].

### 4.3. Statistical Analysis

Before statistical analysis, all data were square root transformed, in order to increase the homogeneity of variances (assessed with Bartlett test) [32].

The effects of the different soil managements (SS, RT, CT) and N supplies (30, 90 kg N ha^−1^) on weed seedbank composition were first investigated using Stepwise Discriminant Analysis (SDA). In particular, to understand how different soil and N managements affected weed species presence, data analysis was carried out, for each soil layer (0–0.20 m and 0.20–0.40 m), considering both the different managements separately (Tillage and Nitrogen) and their interaction (Tillage × Nitrogen).

Given a classification variable and several quantitative variables, SDA selects a subset of the quantitative variables for use in discriminating among the classes (SAS Institute Inc., Cary, NC, USA, 2012). Wilks’ lambda statistics was used as multivariate measure of separability [39,40,41] SDA was performed through the STEPDISC procedure of SAS/STAT using the STEPWISE algorithm (SAS Institute Inc. 2012). Significance level to entry and to stay was set at 0.05.

Analysis of variance was also performed, following a 3-way completely randomized experimental design, in order to find statistical differences among main factors (soil depths, tillage systems, and N levels) and their interactions. Duncan’s test was used to compare means resulting from significant factors.

## 5. Conclusions

The effect of different tillage systems and nitrogen supply could not be promptly evident on the emerged flora because the seed bank, thanks to seed longevity, acts as a reservoir for weed infestation [32].

Our results, as a whole, suggest that different tillage systems and, to a lesser extent, different nitrogen supply, produce changes in the seed bank size and composition, along the soil profile. Therefore, the characterization of seed bank in terms of size and species composition can be a useful tool, more sensitive than actual flora, for predicting the medium- and long-term effects on weed flora of minimal soil disturbance and other conservative practices such as low application of inputs promoted by conservation agriculture systems.

## Figures and Tables

**Table 1:** Species recorded in the soil seed bank and their emergence season (1).

Species	Botanical Family	Code	Emergence Season
*Amaranthus retroflexus* L.	Amaranthaceae	AMARE	Spring/Summer
*Anagallis arvensis* L.	Primulaceae	ANGAR	Spring/Summer
*Chenopodium album* L.	Chenopodiaceae	CHEAL	Spring/Summer
*Conyza canadensis* (L.) Cronq.	Asteraceae	ERICA	Spring/Summer
*Fallopia convolvulus* (L.) Á. Löve	Polygonaceae	POLCO	Spring/Summer
*Fumaria officinalis* L.	Papaveraceae	FUMOF	Autumn/Winter
*Galiumaparine* L.	Rubiaceae	GALAP	Autumn/Winter
*Heliotropiumeuropaeum* L.	Boraginaceae	HEOEU	Spring/Summer
*Papaver hybridum* L.	Papaveraceae	PAPHY	Autumn/Winter
*Papaver rhoeas* L.	Papaveraceae	PAPRH	Autumn/Winter
*Phalaris paradoxa* L.	Poaceae	PHAPA	Autumn/Winter
*Picris echioides* L.	Asteraceae	PICEC	Spring/Summer
*Polygonum aviculare* L.	Polygonaceae	POLAV	Autumn/Winter
*Polygonum hydropiper* L.	Polygonaceae	POLHY	Spring/Summer
*Portulaca oleracea* L.	Portulacaceae	POROL	Spring/Summer
*Setaria viridis* (L.) P. Beauv.	Poaceae	SETVI	Spring/Summer
*Silybum marianum* (L.) Gaertn.	Asteraceae	SLYMA	Autumn/Winter
*Sinapis arvensis* L.	Brassicaceae	SINAR	Autumn/Winter
*Solanum nigrum* L.	Solanaceae	SOLNI	Spring/Summer
*Sonchus oleraceus* L.	Asteraceae	SONOLO	Autumn/Winter
*Veronica hederifolia* L.	Plantaginaceae	VERHE	Autumn/Winter

(1) Botanical nomenclature and codes are listed according to “Composite List of Weeds” redacted by Weed Science Society of America and available on-line (http://wssa.net/).

**Table 2:** Effect of soil depth (D, m), tillage system (T), and nitrogen supply (N, Kg ha^−1^) on weed seed bank (1).

Sources of Variation (2)	Total Seeds	Total Seeds Grouped per Emergence Season	
		Spring/Summer	Autumn/Winter
DEPTH (D)	0.3715	0.4758	0.5083
0–0.2	34.6	18.6	16.0
0.2–0.4	32.4	17.5	14.9
TILLAGE (T)	0.0001	0.0001	0.0138
RT	34.5 B	18.7 B	15.8 AB
CT	21.6 C	9.5 C	12.1 B
SS	44.5 A	26.1 A	18.4 A
NITROGEN (N)	0.0730	0.4130	0.0014
30	31.3	18.7	12.6 B
90	35.7	17.4	18.3 A
D × T	0.0143	0.2741	0.0124
D × N	0.2116	0.7854	0.1086
T × N	0.1483	0.0309	0.9413
D × T × N	0.3478	1.2496	0.7693

(1) The data are listed as the square root of the actual values. (2) Within each column and each factor, data followed by different letters are significantly different at 0.01.

**Table 3:** Results of Stepwise Discriminant Analysis carried out on the weed species of the 0 to 0.20 m layer, considering both the different managements separately (Tillage (b) and Nitrogen (c)) and their interaction (Tillage × Nitrogen (a)).

		Stepwise Selection Summary									
Data Set	n	Step	Number In	Entered (1)	Partial R-Square	F Value	Pr > F	Wilks’ Lambda	Pr < Lambda	Average Squared Canonical Correlation	Pr > ASCC
T × N (a)	18	1	1	ANGAR	0.9888	212.16	<0.0001	0.0111857	<0.0001	0.1977629	<0.0001
		2	2	ERICA	0.9205	25.48	<0.0001	0.00088901	<0.0001	0.3799994	<0.0001
		3	3	HEOEU	0.7988	7.94	0.0029	0.00017884	<0.0001	0.5239311	<0.0001
T (b)	18	1	1	ERICA	0.8421	40	<0.0001	0.15789156	<0.0001	0.4210542	<0.0001
		2	2	PAPRH	0.7214	18.13	0.0001	0.04398084	<0.0001	0.7765215	<0.0001
		3	3	SOLNI	0.5031	6.58	0.0106	0.02185504	<0.0001	0.8171384	<0.0001
		4	4	POLCO	0.5371	6.96	0.0098	0.01011633	<0.0001	0.8378164	<0.0001
N (c)	18	1	1	SINAR	0.3471	8.51	0.0101	0.65286837	0.0101	0.3471316	0.0101
		2	2	HEOEU	0.4262	11.14	0.0045	0.37459124	0.0006	0.6254088	0.0006
		3	3	FUMOF	0.3235	6.7	0.0215	0.25340638	0.0002	0.7465936	0.0002
		4	4	CHEAL	0.2815	5.09	0.0419	0.182079	<0.0001	0.817921	<0.0001
		5	5	PHAPA	0.3236	5.74	0.0338	0.12315314	<0.0001	0.8768469	<0.0001
		6	6	PAPRH	0.3393	5.65	0.0367	0.08137076	<0.0001	0.9186292	<0.0001

See Table 1 for weed codes.

**Table 4:** Results of Stepwise Discriminant Analysis carried out on the weed species of the 0.20 to 0.40 m layer, considering both the different managements separately (Tillage (b)—and Nitrogen (c)) and their interaction (Tillage × Nitrogen (a)).

		Stepwise Selection Summary									
Data Set	n	Step	Number In	Entered (1)	Partial R-Square	F Value	Pr > F	Wilks’ Lambda	Pr < Lambda	Average Squared Canonical Correlation	Pr > ASCC
T × N (a)	18	1	1	PAPRH	0.9179	26.84	<0.0001	0.0820707	<0.0001	0.1835859	<0.0001
		2	2	ERICA	0.8335	11.01	0.0005	0.0136665	<0.0001	0.3448919	<0.0001
		3	3	POROL	0.8666	12.99	0.0004	0.0018228	<0.0001	0.4984092	<0.0001
		4	4	PHAPA	0.947	32.13	<0.0001	9.669 × 10^−5^	<0.0001	0.681228	<0.0001
T (b)	18	1	1	PHAPA	0.7692	25	<0.0001	0.2307692	<0.0001	0.3846154	<0.0001
		2	2	PAPRH	0.6623	13.73	0.0005	0.0779221	<0.0001	0.6975881	<0.0001
		3	3	FUMOF	0.531	7.36	0.0073	0.0365476	<0.0001	0.7823072	<0.0001
N (c)	18	1	1	ERICA	0.3875	10.12	0.0058	0.6125042	0.0058	0.3874958	0,0058
		2	2	SONOLO	0.4193	10.83	0.005	0.3557074	0.0004	0.6442926	0.0004

See Table 1 for weed codes.

**Table 5:** Effect of soil depth (D, m), tillage system (T), and nitrogen supply (N, Kgha^−1^) on weed seed bank composition (1).

Sources of Variation	Weed Species (2)															
	Angar	Erica	Polco	Fumof	Galap	Heoeu	Paphy	Paprh	Phapa	Polav	Porol	Setvi	Sinar	Solni	Slyma	Verhe
Depth (D)	0.4722	0.7886	0.0185	0.6736	0.0312	0.3358	0.7651	0.0001	0.8103	0.0206	0.8871	0.3781	0.0821	0.4285	0.5003	0.0051
0–0.2	3.2	5.9	1.6 a	1.4	1.0 a	1.1	1.6	3.0 a	0.9	1.3 a	0.9	1.6	2.5	1.4	1.2	0.2 b
0.2–0.4	3.5	5.9	0.5 b	1.7	0.2 b	0.6	1.8	1.3 b	1.1	0.2 b	0.9	1.1	3.5	1.8	0.9	1.8 a
Tillage (T)	0.0001	0.0001	0.0001	0.0030	0.0002	0.0005	0.0003	0.0001	0.0063	0.3039	0.0001	0.0007	0.0370	0.0027	0.0001	0.1916
SS	2.5 b	6.9 a	3.1 a	0.4 b	1.8 a	2.3 a	3.8 a	1.9 c	0.3 b	1.1	2.0 a	3.0 a	1.9 b	3.0 a	2.9 a	1.4
RT	6.7 a	5.8 b	0.0 b	3.3 a	0.0 b	0.3 b	0.6 b	4.5 a	0.4 b	0.7	0.0 b	0.7 b	3.1 ab	1.5 b	0.0 b	1.3
CT	0.7 c	4.9 c	0.0 b	1.0 b	0.0 b	0.0 b	0.7 b	0.0 b	2.3 a	0.3	0.6 b	0.3 b	3.8 a	0.3 b	0.3 b	0.3
Nitrogen (N)	0.0001	0.0003	0.4361	0.2071	0.4529	0.3358	0.0357	0.6967	0.6843	0.1569	0.0071	0.1191	0.0001	0.0181	0.1110	0.6105
30	4.6 a	6.3 a	1.2	1.2	0.5	0.6	1.0 b	2.2	0.9	1.0	0.4 b	0.9	1.7 b	2.3 a	0.7	0.9
90	2.1 b	5.5 b	0.9	2.0	0.7	1.1	2.4 a	2.1	1.1	0.4	1.3 a	1.8	4.3 a	0.9 b	1.4	1.1
D × T	0.0506	0.0036	0.0060	0.4835	0.0129	0.0599	0.8414	0.0006	0.0968	0.1303	0.9796	0.2613	0.0525	0.2335	0.8376	0.3034
D × N	0.3551	0.0013	0.1832	0.6365	0.0537	0.0596	0.1031	0.0497	0.6843	0.0206	0.8871	0.5390	0.4526	0.5255	0.0761	0.7365
T × N	0.0007	0.6776	0.5425	0.9952	0.5663	0.0599	0.2317	0.0414	0.5070	0.0268	0.0001	0.0076	0.2879	0.0244	0.3703	0.3034
D × T × N	0.8812	0.0026	0.1747	0.5784	0.0290	0.2038	0.5723	0.3421	0.4170	0.0255	0.9796	0.7650	0.2917	0.1981	0.2557	0.1322

(1) Only species whose data are significant for at least a factor are shown. For the main factors, data within a column followed by different letters are significantly different at the 0.05 *p*-level (Duncan’s Test); (2) See Table 1 for weed codes.

**Table 6:** Main physical and chemical characteristics of the soil in the study area.

Characteristics	Unit	Value
Reaction	pH	7.72
Organic matter (Walkley-Black Method)	%	2.8
Total lime	%	8.8
Sand	%	39.78
Silt	%	37.40
Clay	%	22.82

**Table 7:** Tillage systems and related seedbed preparation and sowing systems used in the experimental field.

Tillage System	Seedbed Preparation	Sowing
Conservative Tillage (SS) = No tillage	Chemical weeding	Sod seeding using direct seed drill machine (“La Seminasodo”)
Reduced Tillage (RT) = Chisel plowing (August)	Disc plowing	Seeder machine “IMA LA Rocca”
Conventional Tillage (CT) = Moldboard plowing (35 cm) (August)		

**Table 8:** Herbicides used for weed management in the crops.

Crop	Herbicides	Doses (L or kg ha^−1^)	Time of Application
Faba bean	Pedimethalin (38.72%)	1.5	Pre-emergence
	Bentazon (87.0%)	0.6	Postemergence (March)
	Fluazifop-*p*-butyl (12.5%)	1.5	
Wheat	Pinoxaden (3.09%)	1.0	
	Clodinafop-propargyl (3.09%)		
	Cloquintocet-mexyl (0.77%)		
Seedbed preparation in sod seeded plots	Glyphosate (48.0%)	3.0	Presowing
